# Functional crosstalk between myeloid Foxo1–β-catenin axis and Hedgehog/Gli1 signaling in oxidative stress response

**DOI:** 10.1038/s41418-020-00695-7

**Published:** 2020-12-07

**Authors:** Changyong Li, Mingwei Sheng, Yuanbang Lin, Dongwei Xu, Yizhu Tian, Yongqiang Zhan, Longfeng Jiang, Ana J. Coito, Ronald W. Busuttil, Douglas G. Farmer, Jerzy W. Kupiec-Weglinski, Bibo Ke

**Affiliations:** 1 The Dumont-UCLA Transplant Center, Division of Liver and Pancreas Transplantation, Department of Surgery, David Geffen School of Medicine at UCLA, Los Angeles, CA USA; 2 Department of Physiology, School of Basic Medical Sciences, Wuhan University, Wuhan, China; 3 Department of Anesthesiology, Tianjin First Center Hospital, Nankai University, Tianjin, China

**Keywords:** Cell death and immune response, Inflammasome, Signal transduction, Acute inflammation

## Abstract

Foxo1 transcription factor is an evolutionarily conserved regulator of cell metabolism, oxidative stress, inflammation, and apoptosis. Activation of Hedgehog/Gli signaling is known to regulate cell growth, differentiation, and immune function. However, the molecular mechanisms by which interactive cell signaling networks restrain oxidative stress response and necroptosis are still poorly understood. Here, we report that myeloid-specific Foxo1 knockout (Foxo1^M-KO^) mice were resistant to oxidative stress-induced hepatocellular damage with reduced macrophage/neutrophil infiltration, and proinflammatory mediators in liver ischemia/reperfusion injury (IRI). Foxo1^M-KO^ enhanced β-catenin-mediated Gli1/Snail activity, and reduced receptor-interacting protein kinase 3 (RIPK3) and NIMA-related kinase 7 (NEK7)/NLRP3 expression in IR-stressed livers. Disruption of Gli1 in Foxo1^M-KO^ livers deteriorated liver function, diminished Snail, and augmented RIPK3 and NEK7/NLRP3. Mechanistically, macrophage Foxo1 and β-catenin colocalized in the nucleus, whereby the Foxo1 competed with T-cell factor (TCF) for interaction with β-catenin under inflammatory conditions. Disruption of the Foxo1–β-catenin axis by Foxo1 deletion enhanced β-catenin/TCF binding, activated Gli1/Snail signaling, leading to inhibited RIPK3 and NEK7/NLRP3. Furthermore, macrophage Gli1 or Snail knockout activated RIPK3 and increased hepatocyte necroptosis, while macrophage RIPK3 ablation diminished NEK7/NLRP3-driven inflammatory response. Our findings underscore a novel molecular mechanism of the myeloid Foxo1–β-catenin axis in regulating Hedgehog/Gli1 function that is key in oxidative stress-induced liver inflammation and necroptosis.

## Introduction

Oxidative stress plays an important role in the pathogenesis of hepatic ischemia and reperfusion injury (IRI), in which IR activates liver macrophages (Kupffer cells) to generate reactive oxygen species (ROS), leading to sterile inflammation and tissue damage [1, 2]. Macrophages are critical mediators of innate immune responses by recognizing exogenous danger signals, such as pathogen-derived molecular patterns or damage-associated molecular patterns (DAMPs) that are released from injured or dying cells during inflammatory response [3, 4]. We have shown that hepatic IR activates liver macrophages and induces toll-like receptor 4 (TLR4) or NLRP3 activation, which drives the innate immunity-mediated liver inflammation [5–8]. Indeed, NLRP3 is regulated by NIMA-related kinase 7 (NEK7), an essential mediator [9], for the initiation of profound sterile inflammatory injury [10, 11].

The Hedgehog signaling pathway is one of the key regulators of cell differentiation, tissue development, homeostasis, and regeneration in mammals [12, 13]. Two essential proteins are involved in Hedgehog signaling activation that includes the G-protein-coupled receptor smoothened (SMO) and the 12-pass transmembrane protein patched 1 (PTCH1). In the absence of Hedgehog ligand, PTCH1 inhibits SMO activation, but when Hedgehog signaling is activated upon binding of the ligand to PTCH1, which leads to SMO activates the glioma-associated oncogene proteins (GLIs). The GLIs then translocate to the nucleus as full-length, activated GLI2 and GLI3 proteins, which induce expression of Hedgehog target genes, including the transcriptional activator Gli1 [14]. Increasing Gli1 activity inhibits proinflammatory mediators, whereas deletion of Gli1 promotes the immune cell activation and tissue inflammation [15]. Under oxidative stress conditions, activation of the Hedgehog/Gli1 signaling reduces cell apoptosis and increases cell survival [16, 17]. Moreover, disruption of the Hedgehog/Gli signaling impairs regeneration of the injured livers [18], suggesting that Hedgehog/Gli pathway may play an important role in the control of liver inflammation and repair during liver injury.

The Foxo subfamily of forkhead (Fox) transcription factors regulates multiple transcriptional targets involved in various cellular processes, including cell survival, apoptosis, differentiation, metabolism, and stress response [19]. Oxidative stress induces Foxo1 nuclear translocation via a c-Jun N-terminal kinase (JNK)-dependent pathway [20]. Increasing Foxo1 activity promotes innate TLR4-mediated inflammatory response and tissue injury [21, 22]. Disruption of Foxo1 increases cardiomyocyte survival and reduces cell apoptosis in response to oxidative stress [23]. Although these studies document important regulatory role of Foxo1 in oxidative stress and inflammatory responses, it is unknown whether and how myeloid Foxo1 may regulate NEK7/NLRP3-driven inflammation and receptor-interacting protein kinase 3 (RIPK3)-mediated necroptosis in IR-triggered liver inflammation.

Here, we identified a novel regulatory mechanism of myeloid Foxo1 signaling on the NEK7/NLRP3 activation and hepatocyte necroptosis in liver IRI. We demonstrated that myeloid Foxo1 deficiency controlled NLRP3 function and alleviated IR-induced hepatocellular injury by inhibiting the Foxo1–β-catenin axis, which in turn enhanced β-catenin/T-cell factor (TCF) binding, promoted the Hedgehog/Gli1 signaling, and activated Snail, leading to reduced NEK7/NLRP3-driven inflammatory response and RIPK3-mediated necroptosis.

## Materials and methods

### Animals

The floxed Foxo1 (Foxo1^FL/FL^) mice (The Jackson Laboratory, Bar Harbor, ME) and the mice expressing Cre recombinase under the control of the lysozyme 2 (Lyz2) promoter (LysM-Cre; The Jackson Laboratory) were used to generate myeloid-specific Foxo1 knockout (Foxo1^M-KO^) mice. Two steps were used to generate Foxo1^M-KO^ mice. First, a homozygous loxP-flanked Foxo1 mouse was mated with a homozygous Lyz2-Cre mouse to generate the F1 mice that were heterozygous for a loxP-flanked Foxo1 allele and heterozygous for the Lyz2-Cre. Next, these F1 mice were backcrossed to the homozygous loxP-flanked Foxo1 mice, resulting in the generation of Foxo1^M-KO^ (25% of the offspring), which were homozygous for the loxP-flanked Foxo1 allele and heterozygous for the Lyz2-Cre allele (Supplementary Fig. 1). The generation of myeloid-specific β-catenin knockout (β-catenin^M-KO^) mice, as described [5]. Mouse genotyping was performed by using a standard protocol with primers described in the JAX Genotyping protocols database. Male C57BL/6 wild-type (WT) mice were obtained from The Jackson Laboratory. Animals at 6–8 weeks of age were used in all experiments. This study was performed in strict accordance with the recommendations in the Guide for the Care and Use of Laboratory Animals published by the National Institutes of Health. The study protocols were approved by the Institutional Animal Care and Use Committee of The University of California at Los Angeles.

### Mouse liver IRI model

We used an established mouse model of warm hepatic ischemia followed by reperfusion [5]. Mice were injected with heparin (100 U/kg), and an atraumatic clip was used to interrupt the arterial/portal venous blood supply to the cephalad liver lobes. After 90 min of ischemia, the clip was removed, and mice were sacrificed at 6 h of reperfusion. Some animals were injected via tail vein 4 h prior to ischemia with an AlexaFluor488-labeled nonspecific (NS) control siRNA or Gli1 siRNA (2 mg/kg; Santa Cruz Biotechnology, CA) mixed with mannose-conjugated polymers (Polyplus transfection™, Illkirch, France) at a ratio according to the manufacturer’s instructions, as previously described [5, 6].

### Hepatocellular function assay

Serum alanine aminotransferase (sALT) levels, an indicator of hepatocellular injury, were measured by IDEXX Laboratories (Westbrook, ME).

### Histology, immunohistochemistry, and immunofluorescence staining

Liver sections (5 μm) were stained with hematoxylin and eosin (H&E). The severity of IRI was graded, using Suzuki’s criteria [24] on a scale from 0 to 4. Liver macrophages were detected, using primary CD11b^+^ rat monoclonal antibodies (mAb; Abcam, Cambridge, MA) and secondary Cy5-conjugated AffiniPure donkey anti-rat IgG (Jackson Immunoresearch, West Grove, PA) for immunofluorescence staining. DAPI was used for nuclear counterstaining. Liver neutrophils were detected by immunohistochemistry (IHC) staining, using primary Ly6G rat mAb (Invitrogen, San Diego, CA). The RIPK3 was analyzed in the liver sections by IHC, using primary RIPK3 mouse mAb (Santa Cruz Biotechnology, Santa Cruz, CA) and normal mouse IgG (Santa Cruz Biotechnology), as an isotype control. Double immunofluorescence staining of RIPK3 and Kupffer cells or hepatocytes was analyzed in the liver sections, using primary RIPK3 mouse mAb (Santa Cruz Biotechnology), CD68 rat mAb (Bio-Rad, Hercules, CA), and HNF-4α rabbit mAb (Abcam). The secondary AlexFluor488-conjugated AffiniPure donkey anti-mouse IgG Ab, Cy5-conjugated AffiniPure donkey anti-rat IgG, and Cy5-conjugated AffiniPure donkey anti-rabbit IgG (Jackson Immunoresearch) were used according to the manufacturer’s instructions. To identify in vivo mannose-mediated delivery of siRNA into macrophages, AlexaFluor488-labeled siRNA (Qiagen, Chatsworth, CA) were mixed with mannose-conjugated polymers and injected via tail vein. Livers were collected at 4 h after injection. Liver sections were stained with rat anti-mouse CD68 antibody (Bio-Rad) and Cy5-conjugated AffiniPure donkey anti-rat IgG antibody (Jackson Immunoresearch). The primary β-catenin rabbit mAb (Cell Signaling Technology, Danvers, MA), Foxo1 mouse mAb (Cell Signaling Technology), Gli1 mouse mAb (Novus Biologicals, Centennial, CO), NLRP3 rabbit mAb (Novus Biologicals), and RIPK3 mouse mAb (Santa Cruz Biotechnology), the secondary AlexFluor488-conjugated AffiniPure donkey anti-mouse IgG, Cy5-conjugated AffiniPure donkey anti-rabbit IgG, and AlexFluor488-conjugated AffiniPure donkey anti-rabbit IgG (Jackson Immunoresearch) were used for staining Foxo1, β-catenin, Gli1, or NLRP3-positive cells. Hepatocyte p-MLKL (mixed lineage kinase domain-like) was detected using primary p-MLKL rabbit mAb (Cell Signaling Technology) and the secondary Cy5-conjugated AffiniPure donkey anti-rabbit IgG (Jackson Immunoresearch), according to the manufacturer’s instructions. Images for immunofluorescence staining were captured using a fluorescence microscope (Keyence BZ-X810, Osaka, Japan), and analyzed using Image-pro Plus software. Positive cells were counted blindly in 10 HPF/section (×200).

### Myeloperoxidase activity assay

The presence of myeloperoxidase (MPO) was used as an index of hepatic neutrophil accumulation. The change in absorbance was measured spectrophotometrically at 655 nm. One unit of MPO activity was defined as the quantity of enzyme degrading 1 μmol peroxide/min at 25 °C per gram of tissue.

### Quantitative RT-PCR analysis

Quantitative real-time PCR (RT-PCR) was performed as described [7]. Total RNA was purified from liver tissue or cell cultures using RNeasy Mini Kit (Qiagen), according to the manufacturer’s instructions. Reverse transcription to cDNA was performed by using SuperScript III First Strand Synthesis System (Invitrogen). Quantitative RT-PCR was performed using the QuantStudio 3 (Applied Biosystems by ThermoFisher Scientific, Waltham, MA). In a final reaction volume of 25 μl, the following were added: 1× SuperMix (Platinum SYBR Green qPCR Kit; Invitrogen) cDNA and 10 μM of each primer. Amplification conditions were: 50 °C (2 min), 95 °C (5 min), followed by 40 cycles of 95 °C (15 s) and 60 °C (30 s). Primer sequences used for the amplification of TNF-α, IL-1β, IL-6, CXCL-10, iNOS, and HPRT are shown in Supplementary Table 1. Target gene expressions were calculated by their ratios to the housekeeping gene HPRT.

### Western blot analysis

Protein was extracted from liver tissue or cell cultures as described [7]. Protein was extracted from liver tissue or cell cultures with ice-cold protein lysis buffer (50 mM Tris, 150 mM NaCl, 0.1% sodium dodecyl sulfate, 1% sodium deoxycholate, and 1% Triton-100). The buffer contains 1% proteinase and phosphatase inhibitor cocktails (Sigma-Aldrich, St. Louis, MO). Proteins (30 µg/sample) in SDS-loading buffer (50 mM Tris, pH 7.6, 10% glycerol, and 1% SDS) were subjected to 4–20% SDS–polyacrylamide gel electrophoresis and transferred to nitrocellulose membrane (Bio-Rad). The membrane was blocked with 5% dry milk and 0.1% Tween 20 (USB, Cleveland, OH). The nuclear and cytosolic fractions were prepared with NE-PER Nuclear and Cytoplasmic Extraction Reagents (ThermoFisher Scientific). The Foxo1, p-JNK, JNK, p-Akt, p-β-catenin, β-catenin, Snail, HMGB1, RIPK3, p-MLKL, NLRP3, cleaved caspase-1, Lamin B2, β-actin (Cell Signaling Technology), Gli1 (Santa Cruz Biotechnology), Sonic Hedgehog (Shh), SMO, NEK7, and TCF4 (Abcam) mAbs were used. The membranes were incubated with Abs, and then added Western ECL substrate mixture (Bio-Rad) for imaging with the iBright FL1000 (ThermoFisher Scientific). Relative quantities of protein were determined by comparing to the β-actin expression, using iBright image analysis software (ThermoFisher Scientific).

### Isolation of hepatocytes and liver macrophages

Primary hepatocytes and liver macrophages (Kupffer cells) from Foxo1^FL/FL^ and Foxo1^M-KO^ mice were isolated, as described [5]. In brief, livers were perfused in situ with warmed (37 °C) HBSS solution, followed by a collagenase buffer (collagenase type IV, Sigma-Aldrich). Perfused livers were dissected and teased through 70-μm nylon mesh cell strainers (BD Biosciences, San Jose, CA). Nonparenchymal cells (NPCs) were separated from hepatocytes by centrifuging at 50 × *g* for 2 min three times. NPCs were suspended in HBSS and layered onto a 50%/25% two-step Percoll gradient (Sigma) in a 50-ml conical centrifuge tube and centrifuged at 1800 × *g* at 4 °C for 15 min. Kupffer cells in the middle layer were collected and allowed to attach onto cell culture plates in DMEM with 10% FBS, 10 mM HEPES, 2 mM GlutaMax, 100 U/ml penicillin, and 100 μg/ml streptomycin for 15 min at 37 °C. Nonadherent cells were removed by replacing the culture medium.

### BMM isolation and in vitro transfection

Murine bone-derived macrophages (BMMs) were generated, as described [8]. In brief, bone marrow cells were removed from the femurs and tibias of WT, Foxo1^FL/FL^, Foxo1^M-KO^, β-catenin^FL/FL^, and β-catenin^M-KO^ mice, and cultured in DMEM supplemented with 10% FCS and 15% L929-conditioned medium. Cells (1 × 10^6^/well) were cultured for 7 days and then transfected with CRISPR/Cas9-Gli1 knockout (KO), CRISPR/Cas9-Snail KO, CRISPR/Cas9-RIPK3 KO, CRISPR-Foxo1 activation, CRISPR-RIPK3 activation, or control vector (Santa Cruz Biotechnology). After 24–48 h, cells were supplemented with 100 ng/ml of LPS for additional 6 h.

### Co-culture of macrophages and hepatocytes

BMMs (1 × 10^6^/well) isolated from Foxo1^M-KO^ mice were cultured in a 0.4 μm pore-size transwell insert (Sigma-Aldrich), and transfected with the CRISPR-Snail KO or control vector followed by LPS stimulation. Primary hepatocytes (4 × 10^5^/well) were cultured in a six-well plate. After 24 h, the transwell insert containing LPS-stimulated BMMs were placed into the six-well plate that hepatocytes were initially seeded. Co-cultures were incubated for additional 24 h with or without adding H_2_O_2_ (200 µM) into the six-well plate that hepatocytes were cultured.

### ELISA assay

Murine serum and cell culture supernatants were harvested for cytokine analysis. ELISA kits were used to measure levels of IL-1β (ThermoFisher Scientific) and HMGB1 (Chrondrex, Redmond, WA).

### Statistical analysis

Data are expressed as mean ± SD and analyzed by Permutation *t* test and Pearson correlation. Per comparison two-sided *p* values <0.05 were considered statistically significant. Multiple group comparisons were made using one-way ANOVA followed by Bonferroni’s post hoc test. When groups showed unequal variances, we applied Welch’s ANOVA to make multiple group comparisons. All analyses were used by SAS/STAT software, version 9.4.

### Immunoprecipitation analysis

BMMs from co-culture were lysed in NP-40 lysis buffer (50 mM Tris pH 7.4, 10 mM EDTA, 150 mM NaCl, and 1% NP-40, ThermoFisher Scientific) containing protease inhibitors. The lysates were incubated with Foxo1 antibody or control IgG and protein A/G beads at 4 ^o^C overnight. After immunoprecipitation, the immunocomplexes were washed with lysis buffer three times and analyzed by standard immunoblot procedures.

## Results

### Disruption of myeloid-specific Foxo1 alleviates IR-induced liver injury, and reduces macrophage/neutrophil infiltration and proinflammatory mediators in IR-stressed liver

**Fig. 1:**
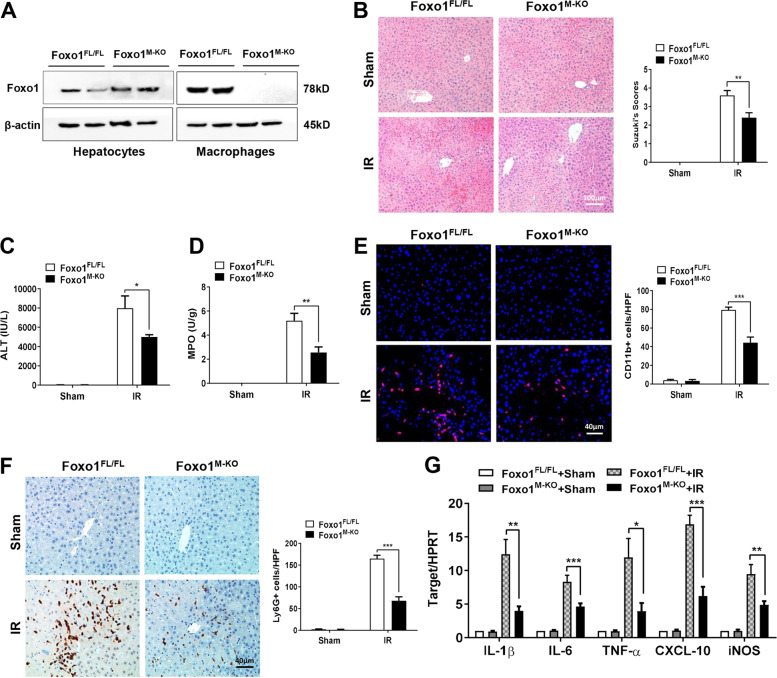
Disruption of myeloid-specific Foxo1 alleviates IR-induced liver injury and reduces macrophage/neutrophil infiltration and proinflammatory mediators in IR- stressed liver. **A** The Foxo1 expression was detected by western blot assay in hepatocytes and liver macrophages from the Foxo1^FL/FL^ and Foxo1^M-KO^ ischemic livers. Representative of three experiments. **B** Representative histological staining (H&E) of ischemic liver tissue (*n* = 4–6 mice/group) and Suzuki’s histological score. Scale bars, 100 μm. **C** Liver function in serum samples was evaluated by serum ALT levels (IU/L; *n* = 4–6 samples/group). **D** Liver neutrophil accumulation, analyzed by MPO activity (U/g; *n* = 4–6 samples/group). **E** Immunofluorescence staining of CD11b^+^ macrophages in ischemic livers (*n* = 4–6 mice/group). Quantification of CD11b^+^ macrophages, Scale bars, 40 μm. **F** Immunohistochemistry staining of Ly6G^+^ neutrophils in ischemic livers (*n* = 4–6 mice/group). Quantification of Ly6G^+^ neutrophils, Scale bars, 40 μm. **G** Quantitative RT-PCR-assisted detection of IL-1β, IL-6, TNF-α, CXCL-10, and iNOS in ischemic livers (*n* = 3–4 samples/group). All data represent the mean ± SD. *p < 0.05, **p < 0.01, ***p < 0.001.

The myeloid-specific Foxo1-deficient (Foxo1^M-KO^) and Foxo1-proficient (Foxo1^FL/FL^) mice were subjected to 90 min of warm ischemia followed by 6 h of reperfusion. We isolated both hepatocytes and liver macrophages (Kupffer cells) from these ischemic livers. Unlike Foxo1^FL/FL^ livers, Foxo1^M-KO^ lacked Foxo1 expression in liver macrophages, but not in hepatocytes (Fig. 1A). The liver damage was evaluated by Suzuki’s histological grading of liver IRI (Fig. 1B). The Foxo1^M-KO^ livers showed mild to moderate edema, sinusoidal congestion, and mild necrosis compared to the Foxo1^FL/FL^ livers, which displayed severe edema, sinusoidal congestion, and extensive hepatocellular necrosis (Fig. 1B). Disruption of myeloid Foxo1 significantly reduced serum ALT levels (IU/L) at 6 h post liver reperfusion in the Foxo1^M-KO^ mice compared to the Foxo1^FL/FL^ controls (Fig. 1C). The MPO levels, which reflect liver neutrophil activity (U/g), were significantly reduced in the Foxo1^M-KO^ group, but not in the Foxo1^FL/FL^ group (Fig. 1D). Moreover, the Foxo1^M-KO^ ischemic livers showed decreased accumulation of CD11b^+^ macrophages (Fig. 1E) and Ly6G^+^ neutrophils (Fig. 1F), accompanied by reduced mRNA levels coding for IL-1β, IL-6, TNF-α, CXCL-10, and iNOS in ischemic livers (Fig. 1G), as compared with the Foxo1^FL/FL^ controls.

### Disruption of myeloid-specific Foxo1 activates the Hedgehog/Gli1 signaling, and inhibits RIPK3 and NEK7/NLRP3 activation in IR-stressed liver

**Fig. 2:**
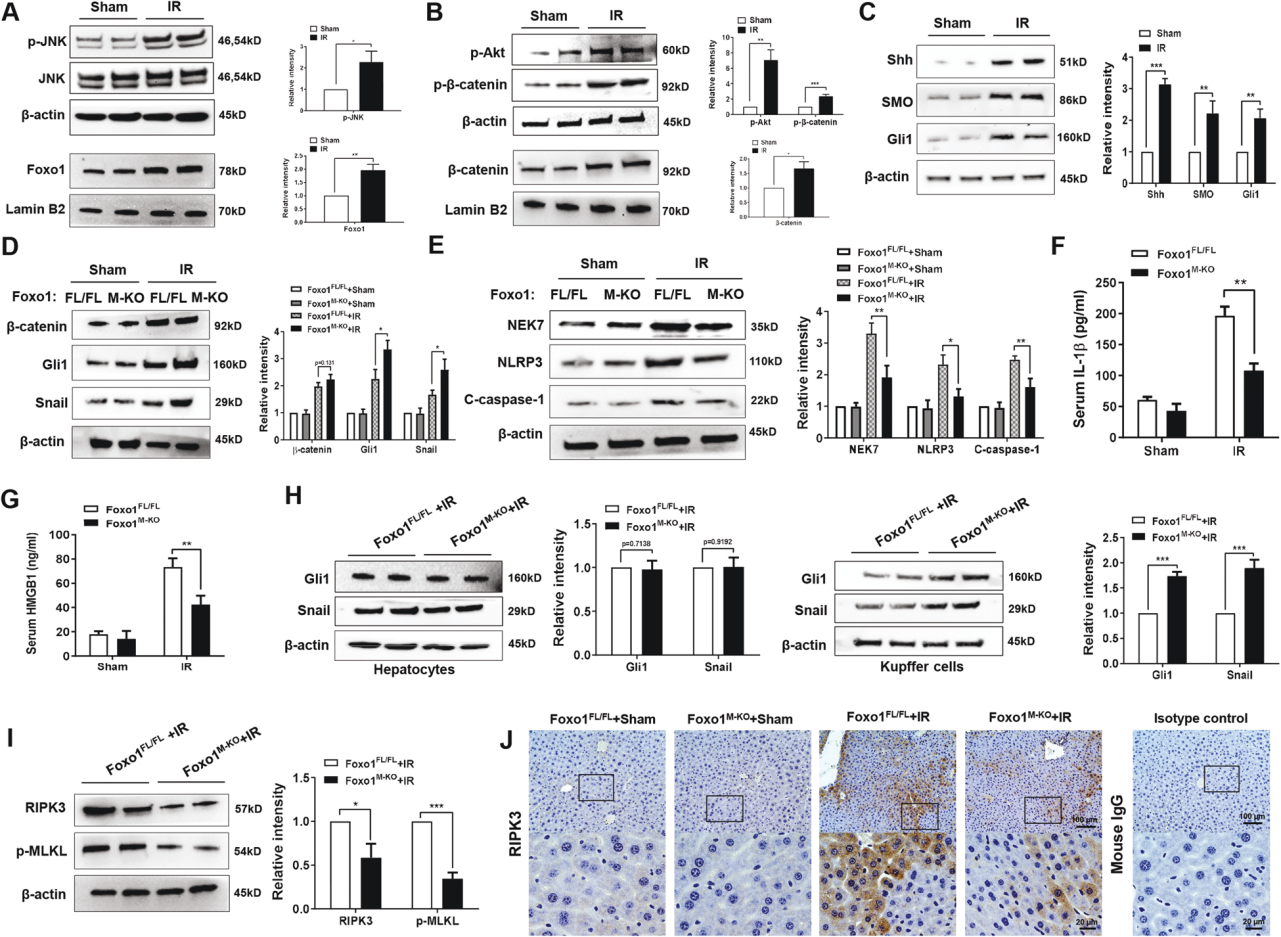
Disruption of myeloid-specific Foxo1 activates the Hedgehog/Gli1 signaling, and inhibits RIPK3 and NEK7/NLRP3 activation in IR-stressed liver. Western-assisted analysis and relative density ratio of **A** p-JNK, Foxo1, **B** p-Akt, p-β-catenin, β-catenin, **C** Shh, SMO, and Gli1 in the WT ischemic livers. Western-assisted analysis and relative density ratio of **D** β-catenin, Gli1, Snail, **E** NEK7, NLRP3, and cleaved caspase-1 in the Foxo1^FL/FL^ and Foxo1^M-KO^ ischemic livers. ELISA analysis of serum IL-1β (**F**) and HMGB1 (**G**) levels in the Foxo1^FL/FL^ and Foxo1^M-KO^ mice after liver IRI (*n* = 3–4 samples/group). **H** The expression of Gli1 and Snail was detected in hepatocytes and Kupffer cells by western blot assay. **I** Western-assisted analysis and relative density ratio of RIPK3 and p-MLKL in the Foxo1^FL/FL^ and Foxo1^M-KO^ ischemic livers. **J** Immunohistochemistry staining of RIPK3 expression in ischemic livers (*n* = 4–6 mice/group). Scale bars, 100 and 20 μm. All western blots represent three experiments and the data represent the mean ± SD. *p < 0.05, **p < 0.01, ***p < 0.001.

Next, we analyzed whether Foxo1 may influence the Hedgehog signaling pathway in IR-stressed livers. Indeed, IR stress induced JNK phosphorylation and increased nuclear Foxo1 (Fig. 2A). IR stress-induced p-Akt phosphorylated β-catenin at Ser552, resulting in augmented nuclear β-catenin expression in ischemic livers (Fig. 2B). Moreover, we found that IR stress activated the Hedgehog/Gli pathway as evidenced by increased expression of Shh, SMO, and Gli1 (Fig. 2C), suggesting that Hedgehog/Gli signaling plays an important role in IR-stressed livers. Furthermore, although there was no significantly change for the Shh and SMO protein expression in IR-stressed livers between Foxo1^M-KO^ and Foxo1^FL/FL^ groups (Supplementary Fig. 2), Foxo1^M-KO^ augmented Gli1 and Snail (Fig. 2D), while inhibiting NEK7, NLRP3, cleaved caspase-1 expression (Fig. 2E), and reduced serum IL-1β levels (Fig. 2F) compared to the Foxo1^FL/FL^ controls. We next measured the levels of HMGB1, an early mediator of sterile inflammatory injury in response to IR stress [25, 26]. Indeed, Foxo1^M-KO^ reduced serum HMGB1 (Fig. 2G) release compared to the Foxo1^FL/FL^ controls. Unlike in hepatocytes, Foxo1^M-KO^ increased Gli1 and Snail expression in Kupffer cells (Fig. 2H). Interestingly, IR stress markedly increased the expression of RIPK3, an essential serine/threonine kinase for necroptosis [27], and its downstream necroptosis executioner MLKL phosphorylation in the Foxo1^FL/FL^ livers. However, Foxo1^M-KO^ diminished RIPK3 and p-MLKL protein levels (Fig. 2I). Consistent with this result, IHC staining also showed reduced RIPK3 expression in the Foxo1^M-KO^ ischemic livers (Fig. 2J). Using double immunofluorescence staining, we further found that reduced RIPK3 expression in both macrophages and hepatocytes in the Foxo1^M-KO^ livers, but not the Foxo1^FL/FL^ controls (Supplementary Fig. 3), suggesting that RIPK3 is involved in necroptotic cell death and liver inflammation after IR stress.

### Hedgehog/Gli1 signaling is required for the regulation of NEK7/NLRP3 and RIPK3 activation in myeloid Foxo1-deficient livers in response to IR stress

**Fig. 3:**
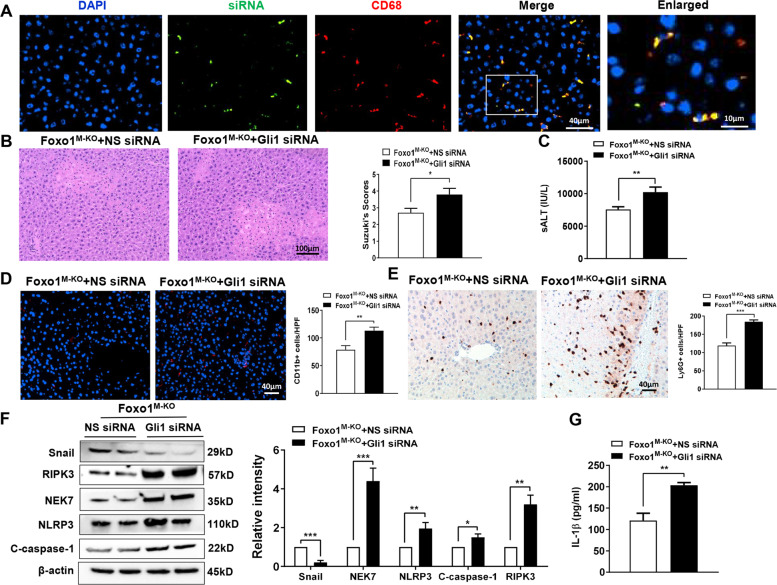
Hedgehog/Gli1 signaling is required for the regulation of NEK7/NLRP3 and RIPK3 activation in myeloid Foxo1-deficient livers in response to IR stress. **A** Immunofluorescence staining of AlexaFluor488-labeled control siRNA and CD68-positive macrophages in ischemic liver tissue (*n* = 3–4 mice/group), Scale bars, 40 and 10 μm. **B** Representative histological staining (H&E) of ischemic liver tissue (n = 4–6 mice/group) and Suzuki’s histological score. Scale bars, 100 μm. **C** Serum ALT levels (IU/L; *n* = 4–6 samples/group). **D** Immunofluorescence staining of CD11b^+^ macrophages in ischemic livers (*n* = 4–6 mice/group). Quantification of CD11b^+^ macrophages, Scale bars, 40 μm. **E** Immunohistochemistry staining of Ly6G^+^ neutrophils in ischemic livers (*n* = 4–6 mice/group). Quantification of Ly6G^+^ neutrophils, Scale bars, 40 μm. **F** Western-assisted analysis and relative density ratio of Snail, NEK7, NLRP3, cleaved caspase-1, and RIPK3. Representative of three experiments. **G** ELISA analysis of serum IL-1β levels (*n* = 3–4 samples/group). All data represent the mean ± SD. *p < 0.05, **p < 0.01, ***p < 0.001.

As myeloid-specific Foxo1 deficiency activated the Hedgehog/Gli1 pathway, we then examined whether Gli1 influenced NEK7/NLRP3 and RIPK3 function in IR-stressed livers. Consistent with previous studies by ours and others [5, 6, 28], we disrupted Gli1 in Foxo1^M-KO^ livers with an in vivo mannose-mediated Gli1 siRNA delivery system that specifically deliver to macrophages by expressing a mannose-specific membrane receptor [29]. Indeed, mannose-mediated AlexaFluor488-labeled siRNA (green) delivery was efficiently transduced into macrophages (red) in IR-stressed livers (Fig. 3A). Knockdown of Gli1 in the Foxo1^M-KO^ mice with the mannose-mediated siRNA treatment aggravated IR-induced liver damage evidenced by increased Suzuki’s histological score (Fig. 3B) and sALT levels (Fig. 3C), compared to the NS siRNA-treated controls. Moreover, Gli1 siRNA treatment in the Foxo1^M-KO^ ischemic livers increased CD11b^+^ macrophage (Fig. 3D) and Ly6G^+^ neutrophil (Fig. 3E) accumulation compared to the NS siRNA-treated controls. Unlike NS siRNA-treated controls, Gli1 siRNA treatment inhibited Snail, but augmented RIPK3, NEK7, NLRP3, and cleaved caspase-1 (Fig. 3F) in the Foxo1^M-KO^ livers accompanied by increased serum IL-1β release (Fig. 3G). Taken together, these data indicate that downstream effector Gli1 of the Hedgehog pathway is essential for the control of RIPK3 and NEK7/NLRP3 function in myeloid Foxo1-deficient livers after IR.

### Foxo1 competes with TCF for interaction with β-catenin and inhibits β-catenin/TCF activity in macrophages

**Fig. 4:**
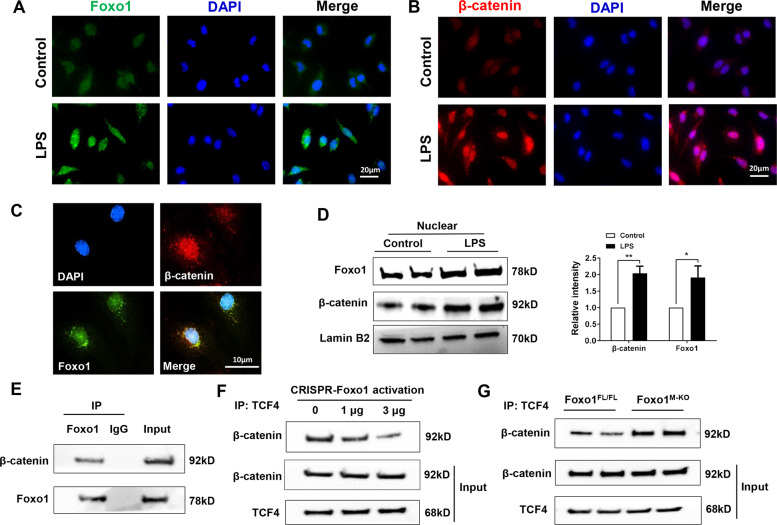
Foxo1 competes with TCF for interaction with β-catenin and inhibits β-catenin/TCF activity in macrophages. Bone marrow-derived macrophages (BMMs) were cultured with or without LPS for 6 h. Immunofluorescence staining of **A** nuclear Foxo1 (green) and **B** β-catenin (red) in LPS-stimulated macrophages. DAPI was used to visualize nuclei (blue). Scale bars, 20 μm. **C** Immunofluorescence staining for macrophage Foxo1 (green) and β-catenin (red) colocalization in the nucleus after LPS stimulation. Scale bars, 10 μm. **D** Western blot-assisted analysis and relative density ratio of nuclear Foxo1 and β-catenin in LPS-stimulated macrophages. Representative of three experiments. **E** Immunoprecipitation analysis of Foxo1 and β-catenin in LPS-stimulated macrophages. Representative of three experiments. **F** BMMs were transfected with CRISPR-Foxo1 activation vector (1 and 3 µg, respectively). Immunoprecipitation analysis of β-catenin and TCF4 in macrophages after LPS stimulation. **G** Immunoprecipitation analysis of β-catenin and TCF4 in LPS-stimulated macrophages from Foxo1^FL/FL^ and Foxo1^M-KO^ mice. All data represent the mean ± SD. *p < 0.05, **p < 0.01.

IR stress increased JNK-dependent Foxo1 transcriptional activity and Akt-mediated β-catenin nuclear translocation leading to regulating the Hedgehog/Gli1 pathway promoted us to investigate the underlying molecular mechanism. We then asked whether there is a putative crosstalk between Foxo1 and β-catenin signaling in the modulation of inflammatory response. Indeed, immunofluorescence staining revealed increased nuclear Foxo1 (Fig. 4A) and β-catenin (Fig. 4B) expression in LPS-stimulated BMMs. Strikingly, both Foxo1 and β-catenin were colocalized in the nucleus (Fig. 4C). To confirm these results, we extracted nuclear protein and analyzed the levels of Foxo1 and β-catenin. As expected, western blot assay showed that increased nuclear Foxo1 and β-catenin protein expression in macrophages after LPS stimulation (Fig. 4D). Next, we used co-immunoprecipitation (Co-IP) assay to detect the Foxo1 and β-catenin interaction under inflammatory conditions. Interestingly, Co-IP clearly showed that Foxo1 bound to endogenous β-catenin in LPS-stimulated macrophages (Fig. 4E), suggesting that Foxo1–β-catenin interaction plays a distinct role during inflammatory response. As the nucleus β-catenin serves as an activator of TCF-dependent transcription leading to modulation of specific target genes [30], we then test whether the Foxo1–β-catenin interaction could influence β-catenin–TCF4 binding. Surprisingly, increased CRISPR-mediated Foxo1 activation reduced nuclear β-catenin–TCF4 binding (Fig. 4F), whereas Foxo1 deletion in macrophages from the Foxo1^M-KO^ mice augmented β-catenin–TCF4 binding after LPS stimulation (Fig. 4G). This data suggests that Foxo1 could control β-catenin/TCF4 complex, thereby regulating β-catenin activity. Taken together, these results indicated that macrophage Foxo1–β-catenin interaction is crucial for the regulation of β-catenin activity during inflammatory response.

### Foxo1 deficiency promotes β-catenin-mediated Gli1 activation and inhibits NEK7/NLRP3-driven inflammatory response in macrophages

**Fig. 5:**
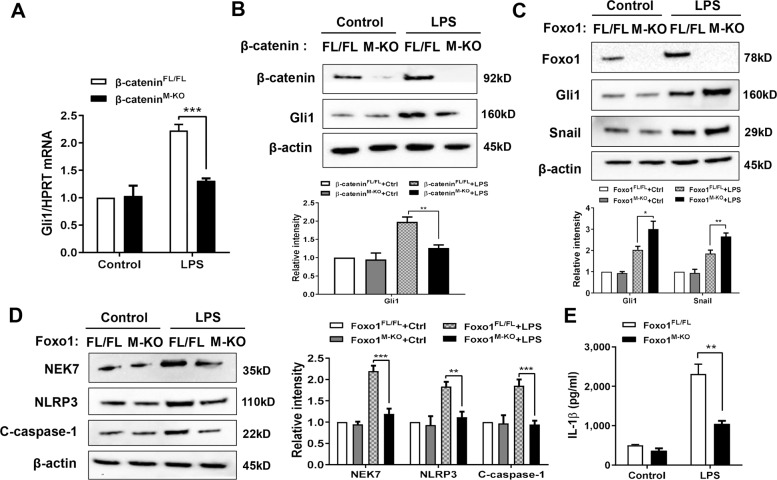
Foxo1 deficiency promotes β-catenin-mediated Gli1 activation and inhibits NEK7/NLRP3-driven inflammatory response in macrophages. BMMs were isolated from Foxo1^FL/FL^, Foxo1^M-KO^, β-catenin^FL/FL^, and β-catenin^M-KO^ mice and incubated with LPS. **A** Quantitative RT-PCR-assisted analysis of Gli1 mRNA in LPS-stimulated BMMs (*n* = 3–4 samples/group). Western blots analysis and relative density ratio of **B** β-catenin and Gli1, **C** Foxo1, Gli1, and Snail, **D** NEK7, NLRP3, and cleaved caspase-1. Representative of three experiments. **E** ELISA analysis of supernatant IL-1β levels (*n* = 3–4 samples/group). All data represent the mean ± SD. *p < 0.05, **p < 0.01, ***p < 0.001.

As transcriptional factor Gli1 is required for the control of RIPK3 and NEK7/NLRP3 function in vivo, we then determine whether the Foxo1–β-catenin interaction influences Gli1 activation in macrophages after LPS stimulation. Using BMMs that were isolated from Foxo1^FL/FL^, Foxo1^M-KO^, β-catenin^M-KO^, and β-catenin^FL/FL^ mice, we found that β-catenin^M-KO^ significantly reduced mRNA level coding for Gli1 in LPS-stimulated macrophages compared to the β-catenin^FL/FL^ controls (Fig. 5A). β-catenin^FL/FL^ showed increased macrophage Gli1 protein expression, whereas β-catenin^M-KO^ displayed reduced expression levels of Gli1 in response to LPS stimulation (Fig. 5B). Moreover, macrophage Foxo1 deficiency augmented Gli1 and Snail (Fig. 5C), accompanied by reduced NEK7, NLRP3, and cleaved caspase-1 expression (Fig. 5D), as well as IL-1β release (Fig. 5E) after LPS stimulation. Therefore, these results suggest that macrophage Foxo1–β-catenin axis plays key role in the modulation of NEK7/NLRP3-driven inflammatory response.

### Gli1 is crucial for the myeloid Foxo1 signaling-mediated immune regulation of RIPK3 and NEK7/NLRP3 activation in macrophages

**Fig. 6:**
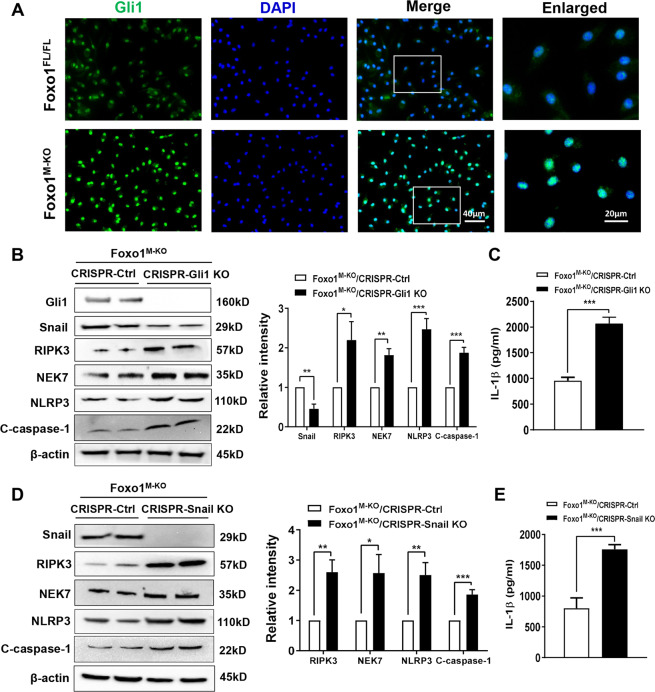
Gli1 is crucial for the myeloid Foxo1 signaling-mediated immune regulation of RIPK3 and NEK7/NLRP3 activation in macrophages. **A** BMMs were isolated from Foxo1^M-KO^ mice and incubated with LPS. Representative immunofluorescence staining for the Gli1 expression in macrophages (*n* = 3–4 samples/group). DAPI was used to visualize nuclei. Scale bars, 40 and 20 μm. **B** BMMs from Foxo1^M-KO^ mice were transfected with p-CRISPR-Gli1 KO or control vector followed by LPS stimulation. Western blots analysis and relative density ratio of Gli1, Snail, RIPK3, NEK7, NLRP3, and cleaved caspase-1. Representative of three experiments. **C** ELISA analysis of supernatant IL-1β levels (*n* = 3–4 samples/group). **D** BMMs from Foxo1^M-KO^ mice were transfected with the p-CRISPR-Snail KO or control vector followed by LPS stimulation. Western-assisted analysis and relative density ratio of Snail, RIPK3, NEK7, NLRP3, and cleaved caspase-1. **E** ELISA analysis of supernatant IL-1β levels (*n* = 3–4 samples/group). Representative of three experiments. All data represent the mean ± SD. *p < 0.05, **p < 0.01, ***p < 0.001.

To dissect the mechanistic role of Gli1 in the regulation of NEK7/NLRP3 activation in the Foxo1–β-catenin axis-mediated immune regulation, we detected the Gli1 expression in BMMs from the Foxo1^FL/FL^ and Foxo1^M-KO^ mice by immunofluorescence staining. Indeed, we found that increased Gli1 expression in the Foxo1^M-KO^, but not the Foxo1^FL/FL^ BMMs after LPS stimulation (Fig. 6A). To examine whether Gli1 may modulate Snail and RIPK3 activation during inflammatory response, BMMs from Foxo1^M-KO^ mice were transfected with CRISPR/Cas9-mediated Gli1 knockout (p-CRISPR-Gli1 KO) or control vector. We found that p-CRISPR-Gli1 KO inhibited Snail but activated RIPK3, which accompanied by increased NEK7, NLRP3, and cleaved caspase-1 expression (Fig. 6B), and IL-1β release (Fig. 6C) compared to the control vector-treated cells. To further tested the mechanistic link between Gli1-mediated Snail and RIPK3 on NEK7/NLRP3 function, BMMs from Foxo1^M-KO^ mice were transfected with p-CRISPR-Snail KO or control vector. Interestingly, unlike control vector-treated cells, transfection of p-CRISPR-Snail KO in the Foxo1^M-KO^ cells markedly increased RIPK3, NEK7, NLRP3, and cleaved caspase-1 expression (Fig. 6D) accompanied by augmented IL-1β release (Fig. 6E). These results demonstrate the essential role of Gli1 for the myeloid Foxo1 signaling-mediated immune regulation of RIPK3 activation and NEK7/RIPK3-driven inflammatory response.

### RIPK3 promotes NEK7/NLRP3 activation in myeloid Foxo1-mediated immune response in macrophages

**Fig. 7:**
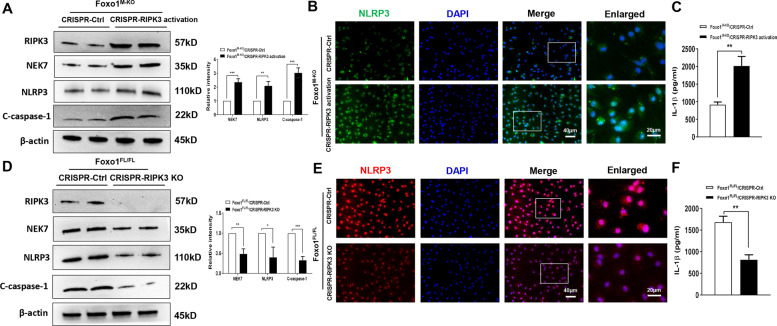
RIPK3 promotes NEK7/NLRP3 activation in myeloid Foxo1-mediated immune response in macrophages. BMMs were isolated from Foxo1^M-KO^ and Foxo1^FL/FL^ mice and transfected with the p-CRISPR-RIPK3 activation, p-CRISPR-RIPK3 KO, or control vector followed by LPS stimulation. **A**, **D** Western blots analysis and relative density ratio of RIPK3, NEK7, NLRP3, and cleaved caspase-1. **B**, **E** Immunofluorescence staining for the NLRP3 expression in macrophages. **C**, **F** ELISA analysis of supernatant IL-1β levels. All western blots represent three experiments, and 3–4 samples each group for immunofluorescence staining and ELISA assay. DAPI was used to visualize nuclei. Scale bars, 40 and 20 μm. The data represent the mean ± SD. *p < 0.05, **p < 0.01, ***p < 0.001.

We next explored whether macrophage RIPK3 was involved in the NEK7/NLRP3-driven inflammatory response. Indeed, transfection of p-CRISPR-mediated RIPK3 activation (p-CRISPR-RIPK3) upregulated NEK7, NLRP3, and cleaved caspase-1 expression in LPS-stimulated BMMs that isolated from the Foxo1^M-KO^ mice compared to the control vector-treated groups (Fig. 7A). Immunofluorescence staining revealed that activation of macrophage RIPK3 by p-CRISPR-RIPK3 transfection increased NLRP3 expression (Fig. 7B) accompanied by augmented IL-1β release (Fig. 7C). In contrast, CRISPR-RIPK3 KO diminished NEK7/NLRP3 and cleaved caspase-1 activation (Fig. 7D, E), and IL-1β release (Fig. 7F) in the Foxo1^FL/FL^ BMMs after LPS stimulation. These results indicate that macrophage RIPK3 triggers NEK7/NLRP3 activation in myeloid Foxo1-mediated immune response.

### Myeloid Foxo1 signaling-mediated Snail regulates RIPK3-mediated hepatocyte necroptosis during inflammatory response

**Fig. 8:**
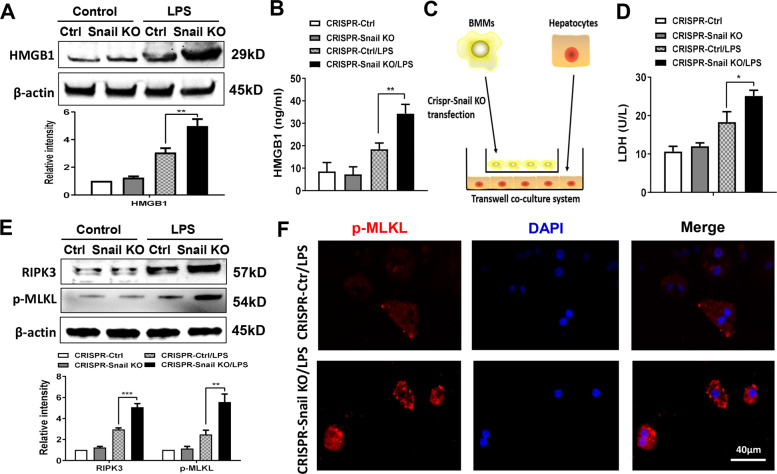
Myeloid Foxo1 signaling-mediated Snail regulates RIPK3-mediated hepatocyte necroptosis during inflammatory response. BMMs were isolated from Foxo1^M-KO^ mice and transfected with the p-CRISPR-Snail KO or control vector followed by LPS stimulation. **A** Western-assisted analysis and relative density ratio of HMGB1 in LPS-stimulated BMMs. Representative of three experiments. **B** ELISA analysis of supernatant HMGB1 levels in LPS-stimulated BMMs (*n* = 3–4 samples/group). **C** Schematic figure for macrophage/hepatocyte co-culture system. **D** BMMs transfected with the p-CRISPR-Snail KO were stimulated with LPS, and then co-cultured with primary hepatocytes that were supplemented with or without H_2_O_2_ for 24 h. LDH release from hepatocytes in co-cultures (*n* = 3–4 samples/group). **E** Western-assisted analysis and relative density ratio of RIPK3 and p-MLKL in hepatocytes after co-culture. Representative of three experiments. **F** Immunofluorescence staining of p-MLKL^+^ hepatocytes after co-culture (*n* = 4–6 mice/group). Scale bars, 40 μm. All data represent the mean ± SD. *p < 0.05, **p < 0.01, ***p < 0.001.

We then asked whether Snail may regulate RIPK3-mediated necroptosis during inflammatory response. As HMGB1 may trigger RIPK3-mediated necroptosis during tissue inflammation and injury [31], we then determined the role of Snail in the regulation of HMGB1 expression during inflammatory response. We found that transfection of Foxo1^M-KO^ BMMs with a p-CRISPR-Snail KO vector markedly increased HMGB1 expression after LPS stimulation, as compared with the control groups (Fig. 8A). Moreover, increased HMGB1 release was observed in the p-CRISPR-Snail KO cells, but not the control cells (Fig. 8B). Using a co-culture system with LPS-stimulated p-CRISPR-Snail KO BMMs from the Foxo1^M-KO^ mice and primary hepatocytes supplemented with H_2_O_2_ (Fig. 8C), we found that macrophage Snail KO markedly increased LDH release from stressed hepatocytes by H_2_O_2_ in the co-culture supernatant (Fig. 8D). Strikingly, unlike the control groups, p-CRISPR-Snail KO augmented hepatocyte RIPK3 and p-MLKL protein levels (Fig. 8E). This was further confirmed by immunofluorescence staining, which showed an increased hepatocyte p-MLKL expression after co-culture with the p-CRISPR-Snail KO BMMs, but not the control cells (Fig. 8F). Taken together, these results indicate that myeloid Foxo1 signaling-mediated Snail is key for the regulation of RIPK3-mediated hepatocyte necroptosis during inflammatory response.

## Discussion

In this study, we document that disruption of myeloid Foxo1 promotes Hedgehog/Gli signaling, and regulates NEK7/NLRP3-mediated immune responses in IR-triggered liver sterile inflammation. Importantly, we show that Foxo1 cooperates with β-catenin signaling to modulate Hedgehog/Gli1/Snail pathway in IR stress mouse liver. First, IR stress induced JNK phosphorylation and increased nuclear Foxo1. Moreover, IR stress activated Akt and promoted nuclear β-catenin translocation. Second, macrophage Foxo1 competed with TCF for interaction with β-catenin in the nucleus and inhibited β-catenin signaling under inflammatory conditions. Third, myeloid Foxo1 deficiency abolished nuclear Foxo1–β-catenin interaction, which in turn enhanced β-catenin activity and promoted Hedgehog/Gli1/Snail signaling, leading to reduced NEK7/NLRP3-driven liver inflammation and RIPK3-mediated necroptosis. Our results highlight the importance of myeloid Foxo1 signaling as a key regulator of the NEK7/NLRP3 activation and hepatocyte necroptosis in IR stress-mediated liver inflammation.

The Foxo1 transcription factor has profound effects in the regulation of innate and adaptive immunity in response to oxidative stress. Foxo1 promotes inflammation by enhancing innate TLR4-mediated pathway in macrophages [21]. Activation of Foxo1 also upregulates dendritic cell (DC) function and activates adaptive immunity [32], whereas disruption of Foxo1 reduces DC-mediated immune response [33]. As a key mediator, Foxo1 promotes proinflammatory cytokine gene program, including monocyte chemotactic protein 1 and IL-6 during inflammatory response [34]. Foxo1 is essential for the mammalian target of rapamycin complex 2-mediated innate immune regulation [35]. Thus, Foxo1 could contribute synergistically and even aggravate innate immune response. Our current study reveals that myeloid Foxo1 deficiency inhibits NEK7/NLRP3 activation and ameliorates IR-induced liver injury, documenting the vital role of the myeloid Foxo1 signaling in modulating NLRP3 inflammasome-mediated inflammatory cascades in IR-stressed livers.

The NLRP3 inflammasome is activated by a number of danger signals [36]. Generation of ROS by oxidative stress is a crucial element for NLRP3 activation [37]. Stress-induced ROS mediates the host defense response by modulating several signaling pathways to induce NLRP3 activation [38]. NEK7 is a key mediator to initiate NLRP3 activation via direct NEK7–NLRP3 interaction in response to ROS [9, 39]. The association between NEK7 and NLRP3 is responsible for inflammasome assembly and the formation of the NLRP3 inflammasome, which results in the activation of caspase-1, secretion, and maturation of IL-1β [40]. Thus, the NEK7–NLRP3 complex might act as an inflammasome sensor during inflammatory response. In line with these findings, we found that disruption of myeloid Foxo1 inhibited NEK7, NLRP3, and cleaved caspase-1 activation, and reduced IL-1β levels, accompanied by increased activation of β-catenin and Gli1/Snail signaling in ischemic livers. These results suggest that β-catenin and Hedgehog/Gli1 signaling are crucial for the myeloid Foxo1 signaling-mediated immune regulation of NEK7/NLRP3 activation in IR-triggered liver inflammation.

The mechanisms underlying myeloid Foxo1 signaling-mediated immune regulation appear to link multiple signaling pathways. Under oxidative stress conditions, Foxo1 changed its intracellular localization from the cytoplasm to the nucleus through activation of the JNK [41]. Inhibition of JNK reduced nuclear localization of Foxo1, suggesting that JNK is essential for the oxidative stress-induced Foxo1 translocation [41]. Consistent with this result, we found that IR stress activated JNK, which in turn stimulated Foxo1 nuclear localization. It is known that Akt signaling pathway is regulated by oxidative stress and is implicated in immunoregulation of innate cells [42]. Activation of Akt by phosphorylation may enhance β-catenin transcriptional activity. Our previous studies demonstrated that the activation of β-catenin reduced TLR4-mediated liver inflammation in a negative feedback regulatory mechanism [43]. The present study revealed that IR stress activated Akt, which phosphorylated β-catenin at Ser552, resulting in translocation of β-catenin into nucleus. This is consistent with previous report that β-catenin activity is dependent on Akt phosphorylation [44]. Nuclearly localized β-catenin interacts with transcription factors of the TCF/LEF, leading to the increased expression of downstream target genes. Thus, we speculate that nuclear localization of endogenous Foxo1 and β-catenin may be essential for the modulation of NEK7/NLRP3 activation in IR-stressed livers. Indeed, using in vitro culture system, we found that macrophage Foxo1 and β-catenin colocalized in the nucleus, and increased nuclear expression of Foxo1 and β-catenin in response to LPS stimulation. Notably, Foxo1 interacted with β-catenin via binding to β-catenin. Increasing Foxo1 activation reduced nuclear β-catenin–TCF4 binding, suggesting a possible regulatory mechanism in which Foxo1 competes with TCF4 for interaction with β-catenin, thereby inhibiting β-catenin activity. Moreover, dissociation of β-catenin from Foxo1 by deleting Foxo1 augmented the β-catenin–TCF4 binding and enhanced β-catenin activity, leading to reduced NEK7/NLRP3 activation in LPS-stimulated macrophages. These results indicate that macrophage Foxo1 displays a distinct ability to control β-catenin activity via the Foxo1–β-catenin axis, which is crucial for the modulation of NEK7/NLRP3 function in response to inflammatory stimulation. Our findings reveal a novel mechanism, in which myeloid Foxo1 signaling regulates NEK7/NLRP3-driven inflammatory response in IR-stressed livers.

The question arises as to what other mechanisms may confer the Foxo1 with the ability to selectively guide the Hedgehog/Gli1 signaling in the modulation of NEK7/NLRP3 function. We have shown that macrophage Foxo1 deficiency enhanced β-catenin signaling via augmenting β-catenin–TCF4 binding. Interestingly, an increased Gli1 expression was observed in β-catenin-proficient macrophages in response to LPS stimulation. However, macrophage β-catenin deficiency diminished Gli1 expression, suggesting the critical role of Hedgehog signaling target gene Gli1 in the modulation of NEK7/NLRP3 function. Further evidence was provided by in vivo study, which showed that disruption of Gli1 reversed myeloid Foxo1 deficiency-mediated cytoprotection, evidenced by augmented IR-induced liver injury and enhanced NEK7/NLRP3 activity. Furthermore, myeloid Foxo1 deficiency increased Gli1 and Snail expression, whereas Gli1 knockdown diminished Snail but augmented RIPK3, indicating that Snail/RIPK3 is regulated by the Hedgehog/Gli1 signaling in IR-stressed liver. Indeed, Snail plays important roles in the modulation of ROS-mediated inflammation. Activation of Snail induces anti-inflammatory cytokines and modulates immune responses [45]. Disruption of Snail signaling increases ROS production and reduced cell survival under oxidative stress [46, 47]. Consistent with these results, we found that disruption of Snail activated RIPK3, an essential serine/threonine kinase for necroptosis [48]. Necroptosis is a highly inflammatory type of cell death as cells can release DAMPs during necroptosis [48]. Indeed, RIPK3-deficient mice displayed reduced inflammatory response and tissue injury in different inflammatory disease models [49]. In line with these findings, we further showed that the activation of RIPK3 enhanced NEK7/NLRP3 function, whereas disruption of RIPK3 inhibited NEK7/NLRP3, resulting in increased caspase-1 activity and IL-1β release in LPS-stimulated Foxo1^M-KO^ macrophages. Our findings reveal an unexpected role for the myeloid Foxo1 signaling by regulating the Hedgehog/Gli1/Snail pathway in controlling RIPK3 function, and NEK7/NLRP3-mediated immune response in IR-triggered liver sterile inflammation.

Another striking finding was that myeloid Foxo1 signaling could be involved in the regulation of IR-induced necroptotic pathways. Under oxidative stress conditions, ROS promotes necroptosis [50], which requires protein RIPK3 and its substrate MLKL. Activation of MLKL by phosphorylation translocates MLKL into the inner leaflet of the plasma membrane and disturbs the integrity of the cell, leading to triggering necroptosis [51]. Thus, RIPK3 and MLKL are essential for contributing to the necroptosis in response to oxidative stress. Current study revealed that myeloid-specific Foxo1 deficiency reduced HMGB1 release, a critical mediator of inflammation, released from activated macrophages and hepatocytes after IR stress. Indeed, HMGB1 can induce RIPK3-mediated necroptosis through activation of TLR4/TRIF signaling [52]. As RIPK3 functions as a key molecule in cell death and survival, we examined RIPK3 expression in vivo. Unlike livers in the Foxo1-proficient mice, which showed increased RIPK3 expression in response to IR stress, myeloid-specific Foxo1 deficiency significantly reduced RIPK3 expression in ischemic livers. Notably, disruption of Gli1 enhanced RIPK3 activation in the Foxo1^M-KO^, but not in the Foxo1^FL/FL^ livers. Moreover, using in vitro macrophage/hepatocyte co-culture system, we observed that disruption of macrophage Snail induced hepatocyte RIPK3 and MLKL activation after co-culture. Further evidence was supported by immunofluorescence staining of hepatocytes after co-culture, which showed that deletion of macrophage Snail increased the hepatocyte p-MLKL expression and LDH release in response to H_2_O_2_-mediated oxidative stress. Taken together, our findings demonstrate that myeloid Foxo1 deficiency-mediated Hedgehog/Gli1/Snail signaling is a key regulator of the RIPK3-mediated hepatocyte necroptosis in IR-stressed livers.

In conclusion, we identify a previously unrecognized role of myeloid Foxo1 in controlling NEK7/NLRP3-driven inflammatory response and RIPK3-mediated necroptosis in IR-stressed livers (Supplementary Fig. 4). The functional interaction between myeloid Foxo1–β-catenin axis and Hedgehog/Gli1 signaling is crucial for the regulation of RIPK3 and NEK7/NLRP3 function during inflammatory response. Therefore, our findings provide the rationale for refined therapeutic strategies for oxidative stress-induced inflammatory diseases, such as liver sterile inflammatory injury.

## Supplementary information

Supplementary materials
