# Supplementary material for: Functional crosstalk between myeloid Foxo1–β-catenin axis and Hedgehog/Gli1 signaling in oxidative stress response
Source: Cell Death Differ. 2020 Dec 7;28(5):1705–19. doi: 10.1038/s41418-020-00695-7 (PMC8167164; doi:10.1038/s41418-020-00695-7)
Supplement: Supplementary file 1 — Supplementary materials [file 41418_2020_695_MOESM1_ESM.docx]

**Supplementary Materials**

**FUNCTIONAL CROSSTALK BETWEEN MYELOID FOXO1-β-CATENIN AXIS AND HEDGEHOG/GLI1 SIGNALING IN OXIDATIVE STRESS RESPONSE**

Running title: Foxo1-β-catenin axis in oxidative stress response

Changyong Li^1,2#^, Mingwei Sheng^1,3#^, Yuanbang Lin^1^, Dongwei Xu^1^, Yizhu Tian^1^, Yongqiang Zhan^1^, Longfeng Jiang^1^, Ana J. Coito^1^, Ronald W. Busuttil^1^, Douglas G. Farmer^1^, Jerzy W. Kupiec-Weglinski^1^, and Bibo Ke^1*^

^1^The Dumont-UCLA Transplant Center, Division of Liver and Pancreas Transplantation, Department of Surgery, David Geffen School of Medicine at UCLA, Los Angeles, CA, USA; ^2^Department of Physiology, School of Basic Medical Sciences, Wuhan University, Wuhan, China. ^3^Department of Anesthesiology, Tianjin First Center Hospital, Nankai University, Tianjin, China

^#^These authors contributed equally to this work.

* **Corresponding author:** Bibo Ke, MD, PhD. The Dumont-UCLA Transplant Center, Division of Liver and Pancreas Transplantation, Department of Surgery, David Geffen School of Medicine at UCLA, 77-120 CHS, 10833 Le Conte Ave, Los Angeles, CA 90095. Tel: (310) 825-7444; Fax: (310) 267-2367; Email: [bke@mednet.ucla.edu](mailto:bke@mednet.ucla.edu).

| **Supplementary Table 1**: Primer sequences for the amplification | | |
| --- | --- | --- |
| Target genes | Forward primers | Reverse primers |
| HPRT | 5’-TCAACGGGGGACATAAAAGT-3’ | 5’-TGCATTGTTTTACCAGTGTCAA-3’ |
| TNF-α | 5’-GCCTCTTCTCATTCCTGCTTGT-3’ | 5’-GATGATCTGAGTGTGAGGGTCTG-3’ |
| IL-1β | 5’-TGTAATGAAAGACGGCACACC-3’ | 5’-TCTTCTTTGGGTATTGCTTGG-3’ |
| CXCL-10 | 5’-GCTGCCGTCATTTTCTGC-3’ | 5’-TCTCACTGGCCCGTCATC-3’ |
| Gli1 | 5’-CCCATAGGGTCTCGGGGTCTCAAA C-3’ | 5’-GGAGGACCTGCGGCTGACTGTGTA A-3’ |
| IL-6 | 5’-GCTACCAAACTGGATATAATCAGGA-3’ | 5’-CCAGGTAGCTATGGTACTCCAGAA-3’ |
| iNOS | 5’-GGTCTTTGAAATCCCTCCTGA-3’ | 5’-AGCTCCTGGAACCACTCGTA-3’ |

**Supplementary Figures and Figure Legends**

**Supplementary Fig. 1**


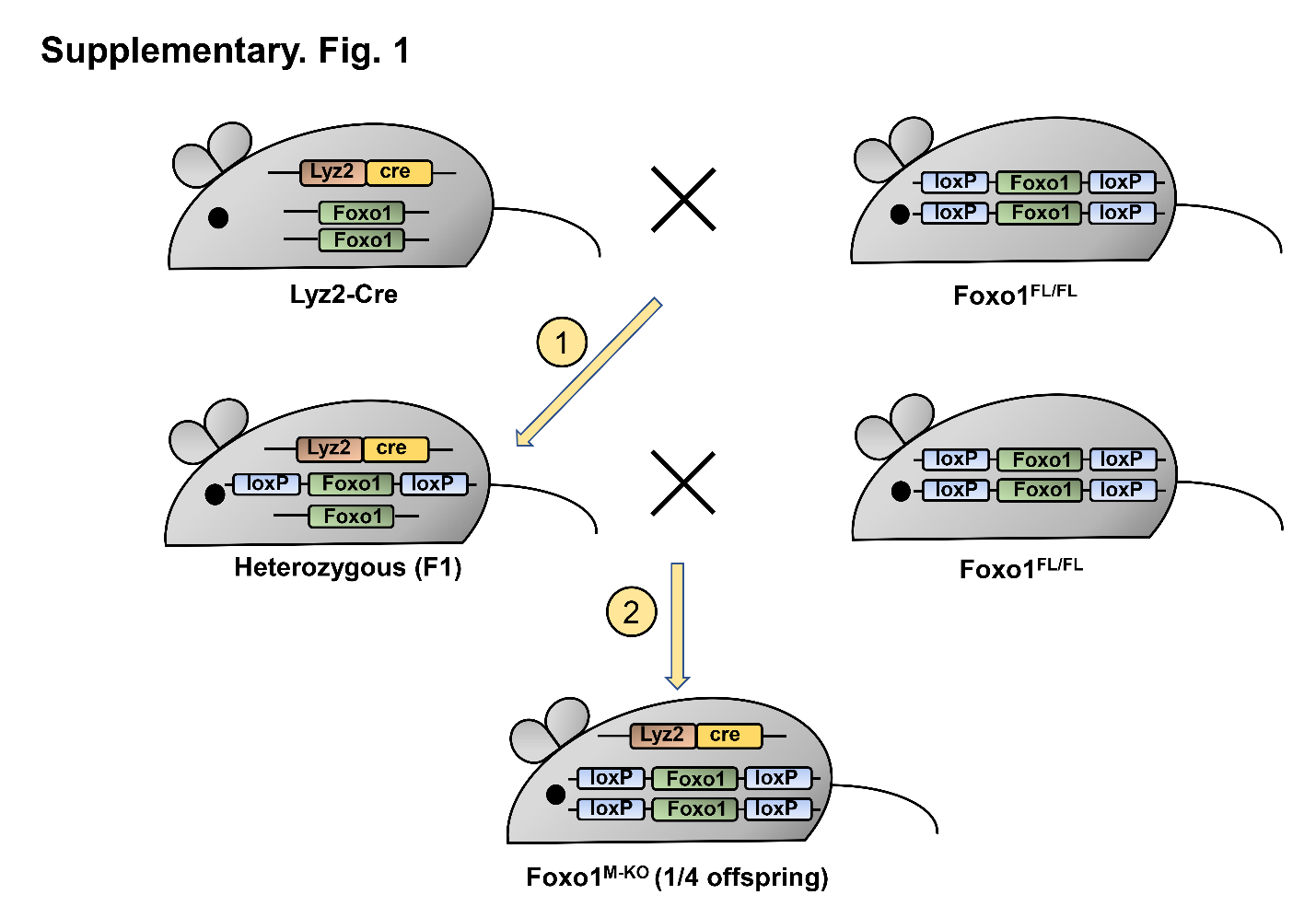


**Suppl. Fig. 1. Schematic illustration of generation of myeloid-specific Foxo1 knockout mice.** Two steps were used to generate myeloid specific Foxo1 KO mice. First, a homozygous loxP flanked Foxo1 mouse is mated with a homozygous Lyz2-Cre mouse to generate the F1 mice that are heterozygous for a loxP-flanked Foxo1 allele and heterozygous for the Lyz2-cre. Next, these F1 mice were backcrossed to the homozygous loxP-flanked Foxo1 mice, resulting in generation of myeloid specific Foxo1 KO mice (Foxo1^M-KO^, 25% of the offspring), which were homozygous for the loxP-flanked Foxo1 allele and heterozygous for the Lyz2-Cre allele.

**Supplementary Fig. 2**


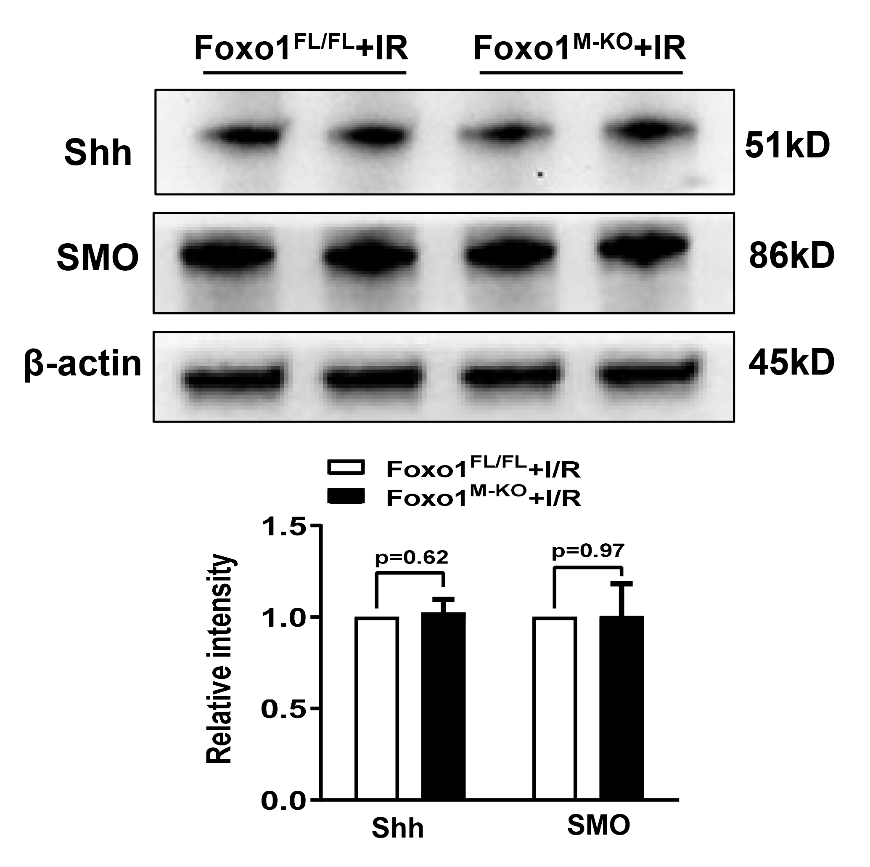


**Suppl. Fig. 2. Myeloid foxo1 deficiency does not alter the expression of Shh and SMO in IR-stressed liver.** The Foxo1^FL/FL^ and Foxo1^M-KO^ mice were subjected to 90min of partial liver warm ischemia, followed by 6h of reperfusion. Western blots analysis and relative density ratio of Shh and SMO. Representative of three experiments. All data represent the mean±SD.

**Supplementary Fig. 3**


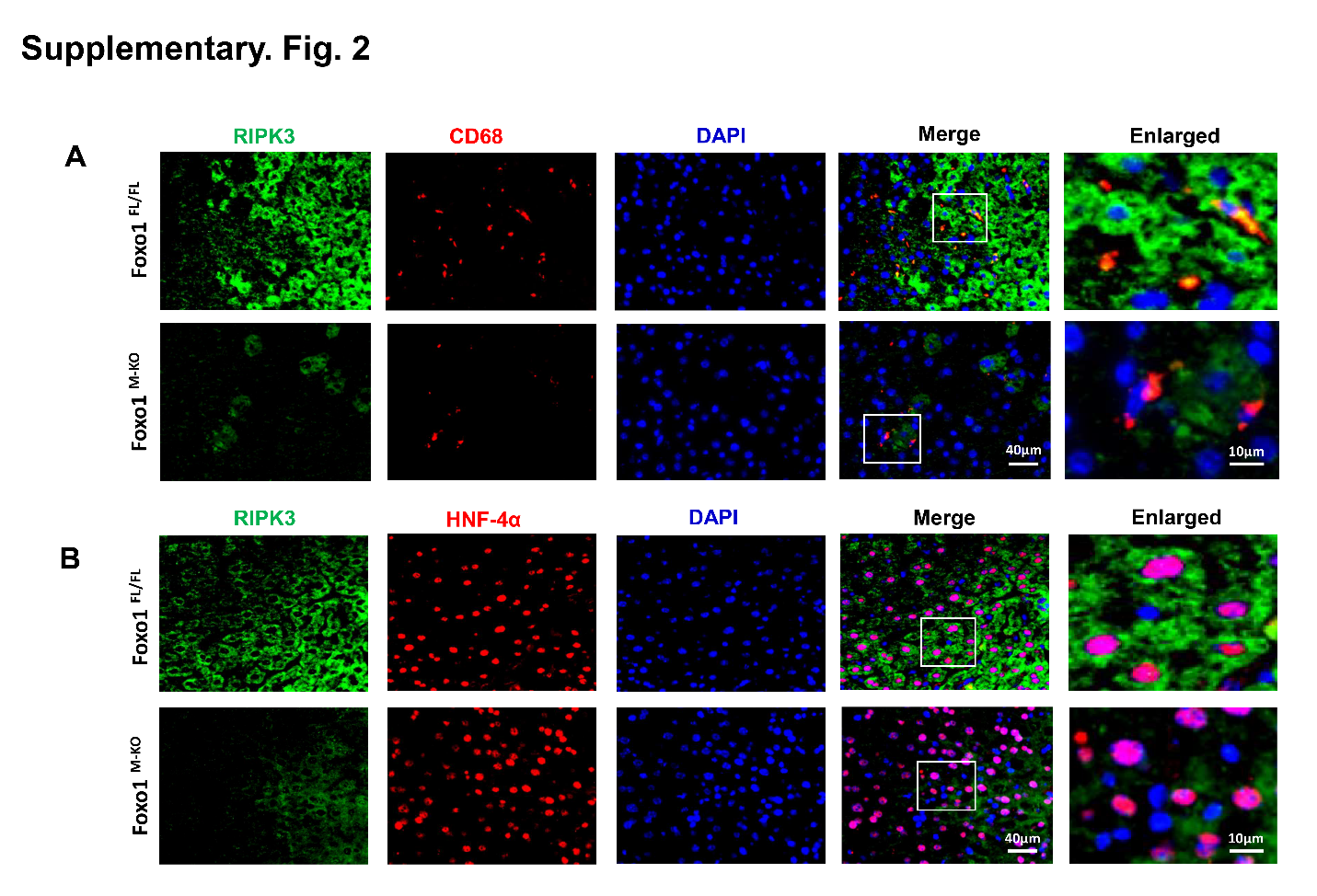


**Suppl. Fig. 3.**  **Disruption of myeloid-specific Foxo1 reduces RIPK3 expression in IR-stressed liver.** The Foxo1^FL/FL^ and Foxo1^M-KO^ mice were subjected to 90min of partial liver warm ischemia, followed by 6h of reperfusion. (A) Representative immunofluorescence staining for RIPK3 expression in macrophages from the Foxo1^FL/FL^ and Foxo1^M-KO^ liver tissues (n=3-4 samples/group). (B) Representative immunofluorescence staining for RIPK3 expression in hepatocytes from the Foxo1^FL/FL^ and Foxo1^M-KO^ liver tissues (n=3-4 samples/group). DAPI was used to visualize nuclei. Scale bars, 40μm and 10μm.

**Supplementary Fig. 4**


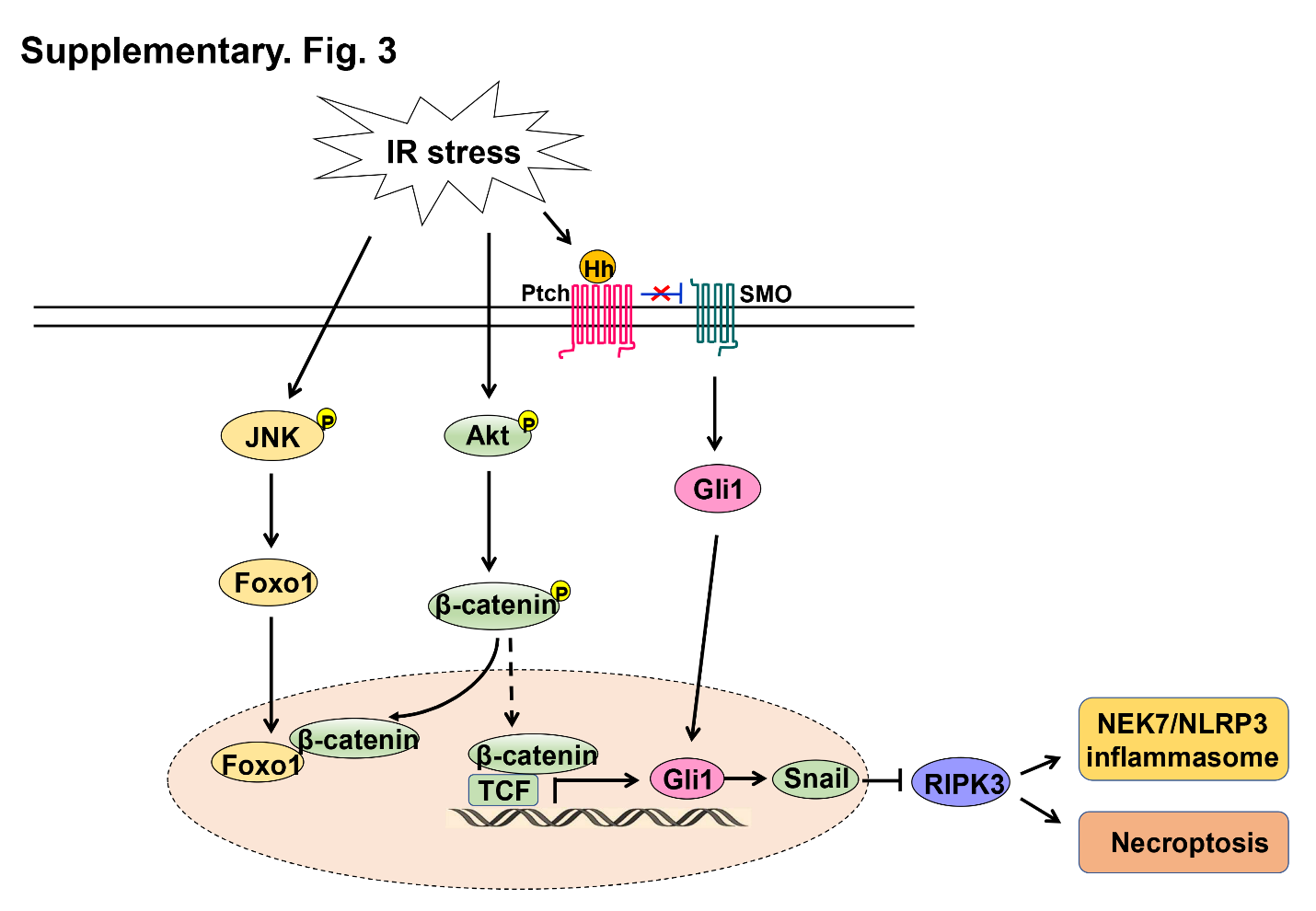


**Suppl. Fig. 4. Schematic illustration of myeloid Foxo1 signaling in the regulation of NEK7/NLRP3 function and RIPK3-mediated necroptosis in oxidative stress-induced inflammation.** IR stress activates JNK and Akt, resulting in increase of Foxo1 and β-catenin translocation from the cytoplasm to the nucleus where Foxo1 competes TCF for interaction with β-catenin. Disruption of the Foxo1-β-catenin axis by Foxo1 deletion enhances β-catenin activity, which in turn promotes Hedgehog/Gli1 signaling and activates Snail leading to diminished RIPK3-mediated NEK7/NLRP3 function and cell necroptosis in oxidative stress-induced inflammation.

**Supplementary Fig. 5**


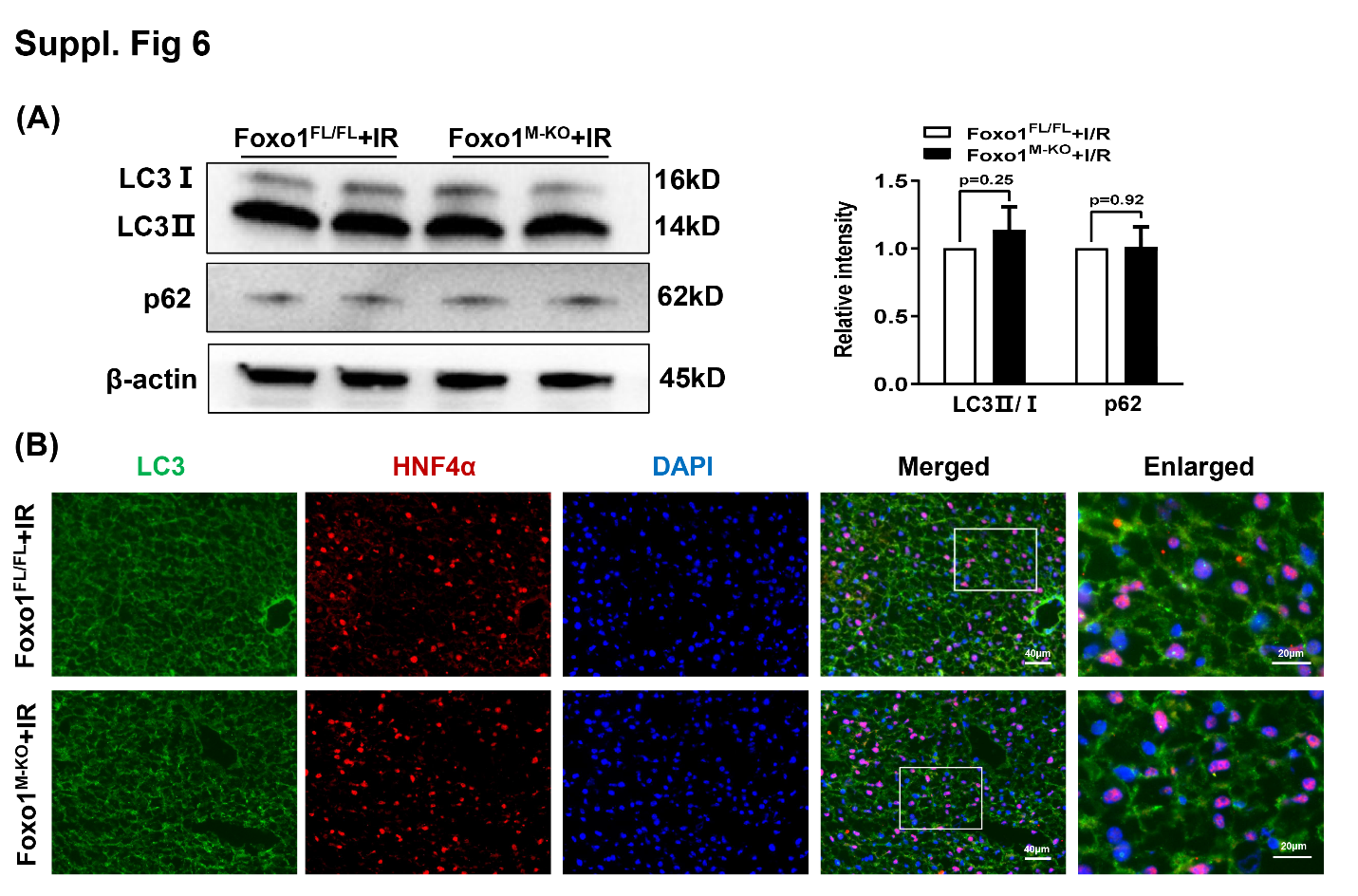


**Suppl. Fig. 5.**  **Disruption of myeloid-specific Foxo1 has no effect on hepatic autophagy in IR-stressed liver.** The Foxo1^FL/FL^ and Foxo1^M-KO^ mice were subjected to 90min of partial liver warm ischemia, followed by 6h of reperfusion. (A) Western blots analysis and relative density ratio of LC3Ⅱ/Ⅰ and p62. Representative of three experiments. (B) Representative immunofluorescence staining for LC3 expression in hepatocytes from the Foxo1^FL/FL^ and Foxo1^M-KO^ liver tissues (n=3-4 samples/group). DAPI was used to visualize nuclei. Scale bars, 40μm and 20μm. All data represent the mean±SD.

Supplementary information is available at *Cell Death & Differentiation*’s website.
